# Constraints on Genome Dynamics Revealed from Gene Distribution among the *Ralstonia solanacearum* Species

**DOI:** 10.1371/journal.pone.0063155

**Published:** 2013-05-28

**Authors:** Pierre Lefeuvre, Gilles Cellier, Benoît Remenant, Frédéric Chiroleu, Philippe Prior

**Affiliations:** 1 CIRAD UMR Peuplements Végétaux et Bioagresseurs en Milieu Tropical, CIRAD-Université de la Réunion, Pôle de Protection des Plantes, Saint Pierre, La Réunion, France; 2 Anses, Laboratoire Santé des Végétaux, Unité Ravageurs et Agents Pathogènes Tropicaux, Pôle de Protection des Plantes, Saint Pierre, La Réunion, France; 3 INRA, Département Santé des Plantes et Environnement, Paris, France; Swiss Federal Institute of Technology (ETH Zurich), Switzerland

## Abstract

Because it is suspected that gene content may partly explain host adaptation and ecology of pathogenic bacteria, it is important to study factors affecting genome composition and its evolution. While recent genomic advances have revealed extremely large pan-genomes for some bacterial species, it remains difficult to predict to what extent gene pool is accessible within or transferable between populations. As genomes bear imprints of the history of the organisms, gene distribution pattern analyses should provide insights into the forces and factors at play in the shaping and maintaining of bacterial genomes. In this study, we revisited the data obtained from a previous CGH microarrays analysis in order to assess the genomic plasticity of the *R. solanacearum* species complex. Gene distribution analyses demonstrated the remarkably dispersed genome of *R.* solanacearum with more than half of the genes being accessory. From the reconstruction of the ancestral genomes compositions, we were able to infer the number of gene gain and loss events along the phylogeny. Analyses of gene movement patterns reveal that factors associated with gene function, genomic localization and ecology delineate gene flow patterns. While the chromosome displayed lower rates of movement, the megaplasmid was clearly associated with hot-spots of gene gain and loss. Gene function was also confirmed to be an essential factor in gene gain and loss dynamics with significant differences in movement patterns between different COG categories. Finally, analyses of gene distribution highlighted possible highways of horizontal gene transfer. Due to sampling and design bias, we can only speculate on factors at play in this gene movement dynamic. Further studies examining precise conditions that favor gene transfer would provide invaluable insights in the fate of bacteria, species delineation and the emergence of successful pathogens.

## Introduction

While some bacteria species display highly monomorphic genomes [1]–[5], some others are highly diverse with genomes bearing numerous imprints of horizontally transferred genes. For the latter, genes histories can be so dramatically different from one another that no linear scenario can properly retrace the history of the whole organism [6], [7].

Genomic analyses of species such as *Neisseria meningitidis* [8]–[9] or *Vibrio cholerae* [10], revealed the existence of extremely large pan-genomes (the set of all genes found in at least one of the genomes, [11]–[14]). In some cases, strains only share half of their gene content with one another, the remaining genes being “accessory” and putatively involved in their lifestyle specificities [15], [16].

Focusing on pathogenic bacteria, analysis of their specialized interactions with animals and plants has demonstrated the involvement of a wide range of evolutionary unrelated enzymatic and biological functions [17]–[21]. In addition to highlighting their tremendous ability to evolve these specialized functions, these analyses have also raised questions on how adaptation factors are acquired and distributed between populations: While a large amount of genes are available in the pan-genome, it remains difficult to predict to what extent these are accessible within or transferable between populations. As gene content bears imprints of the history of the organism, gene distribution pattern analyses should provide insights on the forces and factors at play in the shaping and maintaining of genomes.

*R. solanacearum* [22], a highly destructive and widespread bacterial plant pathogen, is one of the most successful plant pathogens and an excellent model to help answering these questions. This soil-borne xylem inhabitant causes bacterial wilt disease on plants from more than 50 botanical families [23]. *R. solanacearum* is a highly heterogeneous species, both phenotypically and genetically, to which the concept of species complex applies [24], [25]. Previous studies on its genome structure, gene content and distribution [26]–[32] have revealed the remarkable heterogeneity of this bacterial species and the large composition of its pan-genome, to the extent that its classification into different genomic species has been proposed [31]. The *R. solanacearum* species is comprised of four phylotypes that also reflect the region of origin of the isolates, with phylotype I, II, III originating from Asia, America and Africa respectively, while phylotype IV strains originate from Indonesia, Japan and Australia [24], [33].

Interestingly, the genome of *R. solanacearum* is divided in two replicons, a multipartite structure that is associated with its ability to adapt to many different ecological niches with various environmental conditions [34]. Most strains from species belonging to the β-proteobacteria family *Burkholderiaceae,* to which *R. solanacearum* belongs, harbor this multiple replicon structure [35]. As the multipartite genome structure has been maintained throughout the diversification of these organisms, it is likely associated with some selective advantage. The ability to be adapted to multiple lifestyles in various environments is the most shared feature among *Burkholderiaceae*, in particular through interactions (beneficial or parasitic) with eukaryotic organisms [36]–[38]. However, the relationship between the adaptability of the bacterium and the organisation of its genome may not be direct.

In this study, we revisited data collected from multiple comparative genomic hybridization (CGH) microarrays in order to assess the genomic plasticity of the *R. solanacearum* species complex. Based on the reconstruction of the ancestral genome compositions, we were able to infer the number of gene gain and loss along the phylogeny. Analyses of gene movement patterns helped uncover factors limiting gene flow; in particular those associated with gene functions and genome structure.

## Materials and Methods

### Microarray data

The data analyzed here are described in Cellier et al. [39] (available at http://www.ebi.ac.uk/arrayexpress/experiments/E-MTAB-878). Briefly, 72 *R. solanacearum* strains were hybridized onto CGH pan-genomic microarrays. The strains were representative of all the phylotypes of *R. solanacearum* currently described, with most of them (n = 55, 76%) being from phylotype IIB. Hybridization signals were filtered and analyzed to obtain a binary matrix of positive/negative probe signals. Probes were defined so as to be representative of all the CDSs of the six full genomic sequences available at this time. Four of those are considered as “finished” genomes for which gene order is available (GMI1000, CFBP2957, CMR15 and PSI07), while the two remaining genomes are available as scaffolds (Molk2 and IPO1609). From the initial set of 10,762 probes, based on the hybridization properties (inferred using UNAFold; [40]), we decided to trim down the dataset to 7,055 probes for which no ambiguous hybridization results were obtained (no cross hybridization and single target in a genome for each of the probe). We obtained the gene physical location and functions (where available) from the MaGe annotation platform [41]. The sensitivity and specificity of the CGH microarrays were estimated using the recently sequenced R229 and UW551 strains. For both strains we obtained the homologous gene sets with the six fully sequenced strains used to design the arrays from the MaGe annotation platform. While we used different homology cutoffs (ranging from 30% to 99%, Figure S1A), pairwise comparison of the target genes in the six genomes suggested that common target for a probe shares 90% homology or more (Figure S1B). We then compared the actual hybridization profile to the expected profile. Using the 90% homology cutoff, we obtained false positive and false negative rates below 2.1% and 3.7% respectively.

### Phylogenetic reconstruction

The presence/absence signal for each probe allowed reconstruction of the *R. solanacearum* phylogeny using a binary model similar to the F81 nucleotide substitution model, where frequencies and rates of gene gain and loss are estimated independently. In this model, the evolutionary measurable information is the transition between the presence and absence of a probe signal, the changes from one to the other being “probe signal gain” and “probe signal loss” that we considered as “gene gain” and “gene loss”. Because there is a chance that genes are not gained and lost independently due notably to spatial proximity or other codependency factors, we analyzed two distinct datasets. The first was comprised of the whole set of 7,055 probes, whereas the second was composed of a set of 2,992 probes representative of the 2,992 blocks of probes that (1) display the same pattern of presence absence in every strain, and (2) constitute a contiguous physical block in each of the four fully sequence genomes for which gene order is available (GMI1000, CFBP2957, CMR15 and PSI07, [31]). From each of these datasets, a phylogeny was reconstructed using MrBayes v3.2 [42] with the binary model implemented and allowing for variation of substitution rates among sites (selected as best model using the Akaike information criterion). Two runs with four Markov chains were conducted simultaneously for 5,000,000 generations and variations in the likelihood scores were examined graphically with Tracer v1.5 (available at http://tree.bio.ed.ac.uk/software/tracer/). After discarding trees generated prior to convergence of the parameters (burn-in of 10%), consensus phylogeny and posterior probabilities of the nodes were determined. Trees were edited using FigTree v1.3 (available at http://tree.bio.ed.ac.uk/software/figtree/).

### Ancestral character reconstruction and inference of gene gain and loss

To properly infer the gene gain and loss dynamics, we used MrBayes v3.2 [42] to reconstruct the ancestral state of each probe at every node of the phylogeny. In order to control for uncertainty in the tree, including the potential uncertainty concerning the presence of the nodes themselves, an individual analysis was performed for each of the 71 nodes from the 7,055 probe tree. For each node, two runs with four Markov chains were conducted simultaneously for 1,000,000 generations and sampled every 500 generations. After summarizing the sampled trees (with a 10% burn-in), we obtained the probabilities of presence and absence of each gene at every node of the tree. A gene gain was defined as an increase of the probability of presence between two successive nodes of more than 0.5. Conversely, a loss was defined as a decrease of 0.5 in the probability of presence. It was then possible to (1) infer the gene content of the ancestors in the phylogeny; (2) map the events of gene gain and loss on branches and (3) obtain the number of times a single gene was gained and lost.

### Class analysis

Each probe had a specific target in the *R. solanacearum* genome, and some of them were classified by their functions. Of the 7,055 probes, 4,162 were clearly identified and classified in one of the 21 defined COGs [43], while the remaining CDSs code for putative or unknown products. As our dataset contains several genes that have undergone no movement as well as genes that have undergone few movements, we were unable to properly model these distributions, an unavoidable step prior to a statistical parametric analysis. We therefore devised a simple non-parametric permutation-based test. We permuted the COG classification 10^6^ times, summed the gain or loss obtained for each COG and then ranked the sum of each COG from the real dataset within the simulated one. These ranks divided by the number of permutations give a two-tailed *p-*value for having more or less gain and loss than what can be expected by chance. To compare the class dynamics between the chromosome and the megaplasmid, we used a similar test where the probe location was permuted for each COG with *p*-values calculated as described above.

### Spatial analysis

From each of the four fully sequenced and assembled genomes (GMI1000, CFBP2957, CMR15 and PSI07, [31]), probe sets were ordered according to their position on the chromosome and the megaplasmid. The probe order was then permuted 10,000 times but constrained to maintain the integrity of the 2,992 blocks of probes (*i.e.* blocks of contiguous probes that share the exact same patterns of presence/absence). On each of the real and permuted dataset, the gene gain and loss were summed inside a sliding window (size ranging from 100 to 1,000 probes) moved along the genome. The real values were then ranked among the simulated one. These ranks divided by the number of permutation are the two-tailed *p-*value of having a cold-spot or hot-spot of gene movements. *P*-values of 10^−4^ were considered significant. Two tests were devised. In the first “global” test, permutations were performed on both the chromosome and the megaplasmid together, as if they formed a single genomic component, whereas in the second “local test”, permutations were performed on each genomic component independently. The “global test” should provide insights into the relative dynamics associated with the chromosome and the megaplasmid, whereas “the local” test is intended to detect cold-spots and hot-spots of gene gain and loss within each replicon.

The presence of insertion sequences (IS) was assessed using the IS Finder database [44] with default parameters. Hits with e-value superior to 0.05 were discarded. Spatial association of hot-spots and cold-spots of gene movements with IS was tested using the Moran's autocorrelation index implemented in the R [45] package APE [46].

### Horizontal gene transfer

Based on phylogeny and gain/loss data, we were able to reconstruct plausible circuits of gene exchange between individuals. To do so, we focused our analysis on unexpected patterns of gene inheritance. For every pair of strains and ancestral strains (respectively tips and nodes of the tree), we counted the number of genes present in both strains but absent in their most recent common ancestor. A modeling of the linear relationship between these numbers of genes and the genetic distance between strains was first performed using the “lm” (linear model) function available in R before estimating the confidence interval for future outcomes of the model using the “predict.lm” function. Briefly, the prediction provides estimates of the maximum number of newly acquired genes in common between two strains for a given *p*-value threshold. Strains and nodes displaying more genes shared than the 99% confidence interval of the model were hypothesized to be highways of horizontal gene transfer (HGT).

## Results and Discussion

### Phylogenetic reconstruction

**Figure 1:**
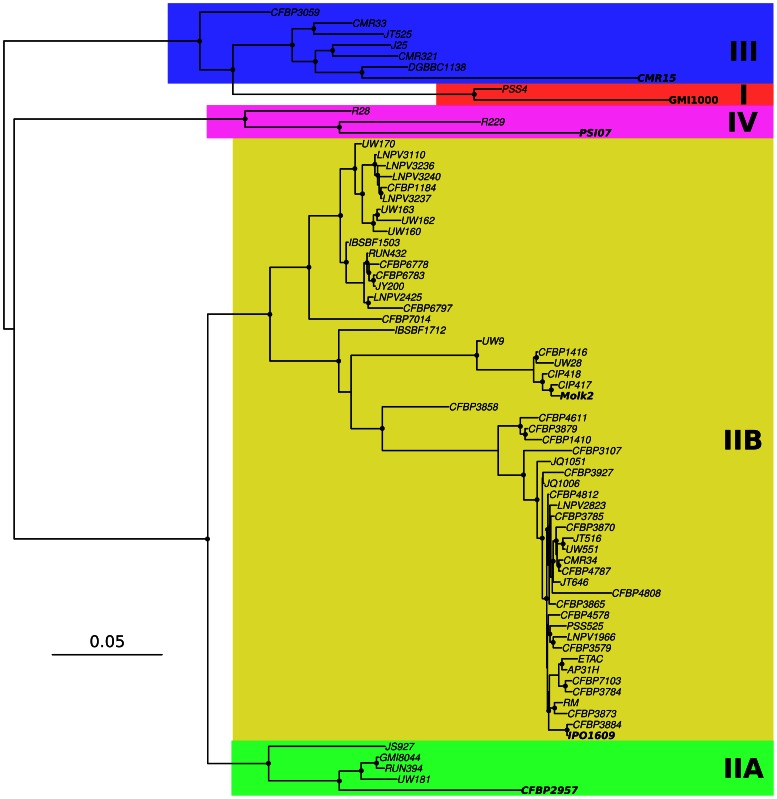
Phylogenetic tree based on the 7,055 probe set. Phylogenetic tree of the *R. solanacearum* species complex inferred using MrBayes and based on the results of the hybridization of 7,055 probes targeting genes from the strains in bold. Phylotype classification is indicated using colored rectangles. Black circles at nodes indicate posterior probability support superior to 95%.

From the CGH microarrays, we reconstructed *R. solanacearum* phylogenies that were highly congruent with phylogenies obtained from *egl* sequencing [47], MLSA [48] or previous CGH studies [29]. Phylogenetic reconstructions based on the 7,055 probes (Figure 1) or on the 2,992 blocks of contiguous probes (Figure S2) presented different basal branching patterns, but the four already described phylotypes were clearly distinct. The sole exception was the position of the CFBP3059 strain that appeared as basal to the closely related phylotypes I and III in the 7,055 probes tree. Although highly congruent grouping of the strains were recovered at the intra-phylotype level from both reconstructions, it is however important to notice that within phylotype IIB, slight differences in branching patterns were observed. These two trees are different views of the evolutionary history of the *R. solanacearum* complex, depending on the unit of evolution being considered: whereas the individual genes themselves are the units of measure in CGH microarray analyses, blocks represent a more parsimonious unit of evolution. The actual *R. solanacearum* phylogeny is probably intermediate between these tree reconstructions.

### Inference of Gene Gain and Loss

**Figure 2:**
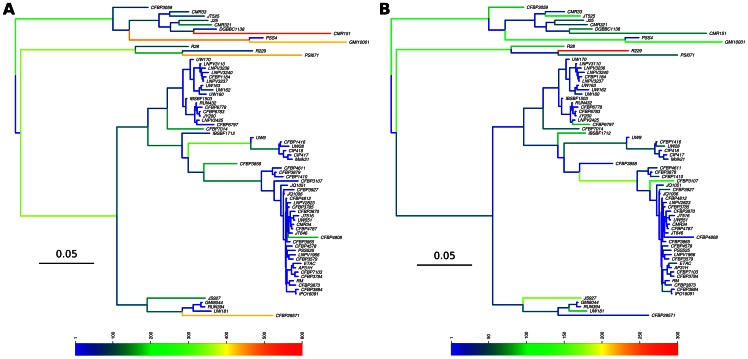
Gene gain and gene loss history. Mapping of the gene gain (A) and gene loss (B) over the *R. solanacearum* phylogeny. Branches are colored according to the number of gain and loss events as per the scale at the bottom.

We reconstructed the ancestral state of every gene at every node of the phylogeny. Using the variation of presence probabilities along the tree, we were able to statistically infer the evolution of gene content through the phylogeny with either gene gain (Figure 2A) or gene loss (Figure 2B). It is important to note that the branch lengths are directly related to the sum of gain and loss that occurred, since it represents our measure of evolution. Distinct patterns of gain and loss were observed across the tree. While some branches displayed high numbers of gene gain (see the orange and red branch along the phylotype I clade for example), others were characterized with many gene losses. This latter case was most pronounced on the branch leading to R229, the banana specific and insect transmitted blood disease bacterium (the red branch on Figure 2B). On this branch, a total of 292 genes distributed in 217 blocks were lost.

These patterns of gain and loss highlight one of the limitations of our design. The microarrays represent a finite repertoire of genes, defined from only six sequenced strains. Therefore, besides uncertainties about using “pan-genomes” as a useful measure [49], our study design is inappropriate when it comes to estimating pan-genome size. However, the high number of genes acquired on the branches connecting these six sequenced strains to their ancestors suggests that the pan-genome of *R.* solanacearum is “open” [14] with each strain presenting several almost strain-specific gained genes. On the other hand, the estimation of the core gene set (at least for those genes present on the microarrays) is probably accurate. Given the number of strains we tested and their diversity, one can expect that genes conserved in all or most of these strains to be present in other yet untested strains.

**Figure 3:**
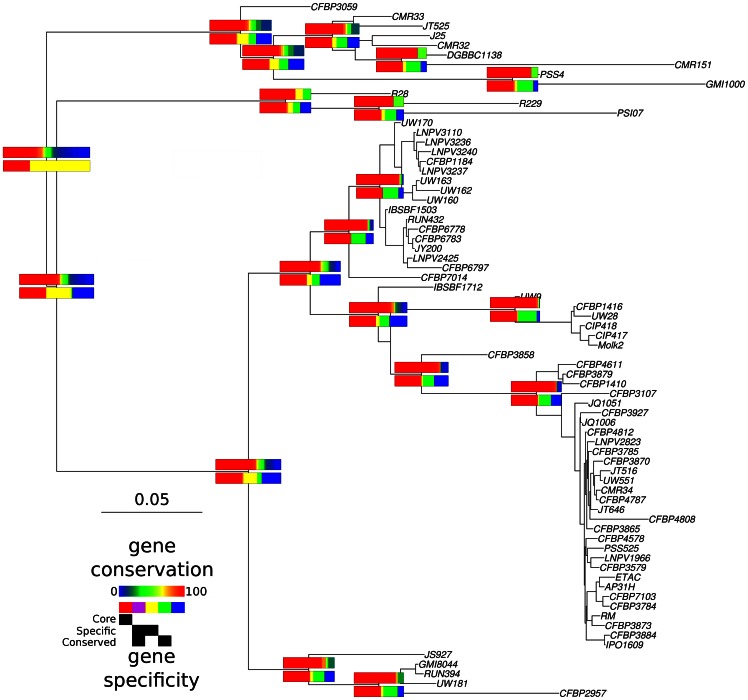
Gene conservation and specificity. Schematic representation of gene conservation and specificity along the phylogeny of the *R. solanacearum* species complex. Only gene content at nodes prior to major splits in the phylogeny are represented. Upper rectangles indicate the degree of gene conservation in the strain to the right of the node with the degree of gene conservation varying from conserved (red) to rare (blue). Lower rectangles indicate the degree of gene conservation in the strain on the right of the node. Red tracks correspond to the proportion of genes from the core genome (conserved in every strain). Purple tracks correspond to specific genes conserved, while yellow tracks indicate specific genes that are not conserved. Non-specific genes are indicated with green (conserved) and blue (not conserved) tracks. The relative length of the rectangles is proportional to the number of genes present in the strains to which they refer.

A total of 2,155 genes (∼30% of the 7,055 tested genes) were present in every genome, while the remaining genes were usually present at low frequency (36% of the genes are present in less than 10% of the strains). The evolution of the gene content for some of the major nodes of the *R. solanacearum* phylogeny is depicted on Figure 3. The upper rectangles at each node represent the degree of conservation of genes present in the group of strains above the node. For the most recent common ancestor of all *R. solanacearum* (*i.e.* the deepest node in the phylogeny), gene frequency presented the so-called “U-shape” distribution (for details, see [50]): genes were either present at high (reddish color) or low frequencies (bluish color) with few genes present at medium frequencies. The gene set appeared more conserved between more recent nodes but there is still a significant proportion of accessory genes. Distribution of genes among clades are represented by the lower rectangles. While the core gene set (colored in red) was by definition stable over the tree, around half of the genome was composed of accessory genes with complex group associations (the other colors). Importantly, there were extremely few genes conserved and specific to a group (purple tracks) that can be mapped to any node. The maximum number of specific and conserved genes was found in the ancestor of the phylotype IV (n = 139) followed closely by the ancestor of phylotype I (n = 132). In contrast, only 99 genes were specific to the phylotype II strains. As the number of tested strains was very different between phylotypes, these numbers are difficult to interpret but they do demonstrate the low specificity of genes at the phylotype level. A larger proportion of specific and non-conserved genes were detected (yellow tracks). This proportion decreases rapidly to a small fraction at the intra-phylotype level. Interestingly, the vast majority of the non-core genes were non-specific to any of the clades (green and blue tracks, for respectively non-core conserved and non-core not conserved genes). This particularity highlights the extreme dispersal of the pan-genome of *R. solanacearum* explained either by frequent HGT between strains or by low frequencies of some of the genes within the populations. In this second hypothesis, gene frequencies may rise and fall within populations depending on a combination of selection and drift.

Because of the clearly distinct phenotypes between strains from phylotype IV (*i.e.* broad host range for *R. solanacearum vs* banana specific strains of the phylotype IV blood disease bacterium [51]), it was anticipated that these ecotypes evolved in isolation and may have developed or acquired a large proportion of specific genes. Conversely, the gene content analysis revealed that this group possessed at least 866 non-specific genes also dispersed among the other phylotypes.

### Differential gene class dynamics

While the CGH experimental design used here doesn't allow us to obtain complete gene contents for the tested strains, we are nevertheless confident in our ability to properly track gene movements. We therefore tested for specific patterns of gene inheritance and transfer. We attempted to determine if there were differences in the dynamics of acquisition/loss of the different clusters of orthologous groups (COG, [43]) by regrouping genes depending on their functions in 21 classes such as “Transcription” and “Cell motility”. For the 4,162 probes for which COG classifications were available, we obtained a sum of gene gain and loss for each of the 21 classes. Then, using a permutation test, the number of genes gained and lost were compared between the different COG classes. The test provided a *p*-value for having more or less gain and loss in a given category than what would be expected by chance.

After a million permutations, it was clearly apparent that COGs were an important factor in determining the mobility of genes (Table 1). Clear signals of non-random gene gains or losses dependent on the COG class were detected. In the “complexity hypothesis” [52], it is suggested that the transferability of genes between genomes is dependent on the biological process and the connectivity of the network a gene is involved in. Whereas, the distinct contributions of connectivity and function in gene transferability were recently revisited [53], summarizing gene dynamics at the COG class level was proved to reveal differential dynamics [54]–[57]. In agreement with these previous studies, and as demonstrated in *Bacillus subtilis* and *Escherichia coli* [54], [55], we detected the “Translation, ribosomal structure and biogenesis” category as the most stable in *R. solanacearum*. It was also interesting that genes involved in “Nucleotide transport and metabolism” appeared to be highly stable with both loss and gain occurring less than what would be expected by chance. Other categories such as “Energy production and conversion” and “Post-translational modification, protein turnover, chaperones” were also stable with less loss or gain than expected by chance. Conversely, genes involved in “Cell motility”, “Transcription”, “Lipid transport and metabolism” and “Secondary metabolite biosynthesis, transport and catabolism” presented high mobilities. These results were particularly interesting as the “Cell motility” COG hosts several type III and type IV effectors genes, which may be involved in host adaptation and differential pathogenesis. Both categories of COG from the “poorly characterized” section were highly gained as was previously demonstrated [56] and expected since those genes are less likely to be involved in housekeeping functions. Interesting patterns were obtained for genes involved in the “Replication, recombination and repair” category where genes were lost less and gained more than expected by chance.

**Table 1:** Gene loss and gain depending on the COG classification and the genomic location.

			Global					Chromosome					Megaplasmid									
				Loss		Gain			Loss		Gain			Loss		Gain		Ch. *vs* Mp.				
Process	COG ID	Description	Gene	nb	*p* $	nb	*p* $	Gene	nb	*p* $	nb	*p* $	Gene	nb	*p* $	nb	*p* $	Gain	p£	Loss	p£	
Information storage and processing	A	RNA processing and modification	2	2		0		2	2		0		0	0		0						
	J	Translation, ribosomal structure and biogenesis	154	43	−	42	−	132	26	-	12	−	22	17		30		ch < mp	***	ch < mp	*	
	K	Transcription	397	272	+	324		235	99		187	+	162	173		137				ch < mp	***	
	L	Replication, recombination and repair	225	66	−	253	+++	174	37	-	181	+++	51	29	-	72	++			ch < mp	*	
Cellular processes and signaling	D	Cell cycle control, cell division, chromosome partitioning	45	13		37		36	7		29		9	6		8						
	M	Cell wall/membrane/envelope biogenesis	245	116		141	-	164	57		73		81	59		68		ch < mp	**	ch < mp	*	
	N	Cell motility	143	96		167	+++	76	42		126	+++	67	54		41	−	ch > mp	***			
	O	Posttranslational modification, protein turnover, chaperones	134	66		56	−	112	44		36	−	22	22		20		ch < mp	*			
	T	Signal transduction mechanisms	169	97		108		98	30		51		71	67		57				ch < mp	***	
	U	Intracellular trafficking, secretion, and vesicular transport	72	38		67		41	11		30		31	27		37						
	V	Defense mechanisms	83	49		66		47	19		43		36	30		23						
Metabolism	C	Energy production and conversion	291	119	-	180		194	37	−	92		97	82		88		ch < mp	***	ch < mp	***	
	E	Amino acid transport and metabolism	565	352		335	−	365	129		161	−	200	223		174		ch < mp	***	ch < mp	***	
	F	Nucleotide transport and metabolism	82	19	−	32	−	70	14		24	-	12	5		8						
	G	Carbohydrate transport and metabolism	139	70		97		97	38		66		42	32		31						
	H	Coenzyme transport and metabolism	105	47		35	−	77	17		11	−	28	30		24		ch < mp	***	ch < mp	***	
	I	Lipid transport and metabolism	203	152	+	122		130	57		57		73	95	+	65		ch < mp	**	ch < mp	***	
	P	Inorganic ion transport and metabolism	164	96		88	-	108	40		50		56	56		38				ch < mp	*	
	Q	Secondary metabolites biosynthesis, transport and catabolism	134	128	+++	145	++	71	45	+	60		63	83		85	++	ch < mp	*	ch < mp	*	
Poorly characterized	R	General function prediction only	450	255		393	++	292	131		236	++	158	124		157				ch < mp	*	
	S	Function unknown	360	205		348	+++	235	77		167		125	128		181	+++	ch < mp	***	ch < mp	***	

$ - p-value<0.025; – p-value <0.01; – p-value <0.001; * p-value>0.975; ** p-value >0.99; *** p-value >0.999

£ * p-value<0.025; ** p-value <0.01; *** p-value <0.001

Gene class dynamics were also different depending on whether genes were located on the chromosome or on the megaplasmid. These differential patterns may in fact be caused by the imbalanced distribution of genes depending on the component localization (Chi-square *p*-value <2.10^−16^): some COGs (such as “Transcription”, “Cell motility” and “Secondary metabolites biosynthesis, transport and catabolism”) were over-presented on the megaplasmid in comparison to the chromosome. It was nevertheless clearly apparent that overall the megaplasmid tends to lose (*p*-value <10^−6^) and to gain (*p*-value <10^−6^) more genes than expected, compared to the chromosome. Regarding the COGs themselves, the megaplasmid tended to present more gain and more loss for almost every COG (Table 1). Interestingly, only the “Cell Motility” class presented more gain on the chromosome than on the megaplasmid. However, this provides additional evidences that each replicon does not contribute equally to the genomic plasticity, adaptability and diversification of *R. solanacearum*.

### Spatial structure of gene dynamics

**Figure 4:**
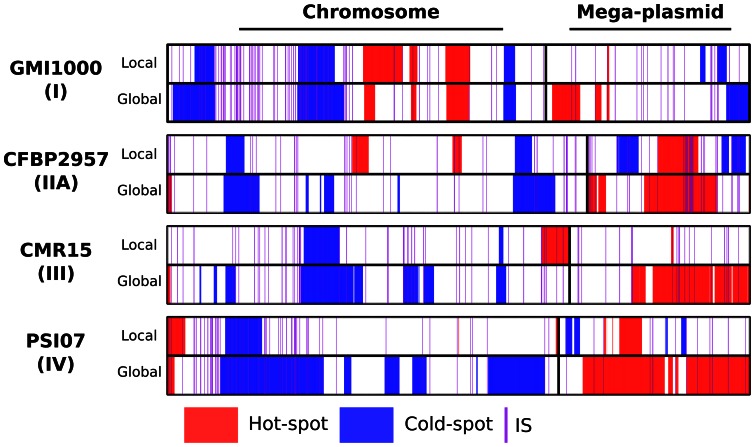
Cold-spots and hot-spots of gene movement. Schematic representation of the cold-spots and hot-spots of gene movement along the genomes of the GMI1000, CFBP2957, CMR15 and PSI07 strains. Cold-spots are indicated in blue while hot-spots are indicated in red. Putative IS elements are represented with purple lines. For every genome, two tests (*p*-value <10^−4^) are represented. For each test, gene movement patterns were compared to those obtained after 10,000 permutations of gene order using a sliding window of 400 genes. In the first “global” test, permutations were performed on the concatenated chromosome and megaplasmid. This test was designed to detect differential patterns between both components. In the second “local” test, permutations were performed on each genomic component separately. The second test was designed to detect intra-component patterns of differential gene movements.

In order to more precisely characterize the differences in gene dynamics between the two genomic components, we tested for the presence of hot-spots and cold-spots of gene movement (*i.e.* the sum of the gene gain and gene loss) in the genome. Using a permutation test, but importantly while taking into account blocks of probe putatively transferred together (*i.e.* the 2,992 blocks), our analysis (Figure 4) confirmed the imbalanced nature of gene movements along the genome with the detection of several cold- and hot-spots. The analysis confirmed the tendency of the megaplasmid to display more gene flow than the chromosome. Most of the cold-spots detected using the global test were on the chromosome, whereas the hot-spots mapped preferentially on the megaplasmid. This was clearly apparent in the CMR15 and PSI07 genomes, using a window size of 1,000 probes (Figure S3 D) to detect global differences in gene movement. Multiple replicon genome organization may be a convenient way to acquire and lose genes without disrupting the whole genome architecture. Although it bears essential genes (since *R. solanacearum* cells cannot survive if they lose their megaplasmid [58]), the megaplasmid may have evolved as a preferential zone for insertion or deletion of genes. Also, because of the high frequency of such events, it is possible that some region of the megaplasmid may be transiently non-functional.

Between two and five local hot-spots of gene movement and between one and five local cold-spots of gene movement (*p*-value <10^−4^) were detected on the chromosome and megaplasmid respectively. These regions varied greatly in length with hot-spots ranging from 1 to 251 genes (mean size of 93 genes) and cold-spots ranging from 2 to 247 genes (mean size of 71 genes).

When analyzing the hot-spots, we didn't detect conservation of the their genomic location or their genomic content between phylotypes. This is in apparent contradiction to previous observations on *E. coli* [59], where regions of integration tended to be conserved. It is thought that after a first successful integration, later integrations would tend to occur at the same place as they may have a lesser impact on the genomic organization. *R. solanacearum* genomes nevertheless bore some imprints of this phenomenon as several combinations of genes were detected in hot-spots from strains of the same phylotype, suggesting repeated and independent integrations and deletions of genes at a similar location. For example, among the 60 strains from phylotype II, up to 49 combinations of genes were associated with a hot-spot (247 genes) detected on the megaplasmid.

We later tested for the association between hot-spots and IS elements using a spatial autocorrelation test. More than 3,400 IS elements from 18 families were detected in the four fully assembled genomes. We used Moran's autocorrelation index with the distance between hot-spot or cold-spot genes to the nearest IS element as a weight matrix. No significant association was obtained, demonstrating that in *R. solanacearum,* IS elements may not drive the cold- and hot-spots clusterings. The same results were obtained when we considered each IS family separately.

### Horizontal gene transfers (HGT) delineate exchange groups

**Figure 5:**
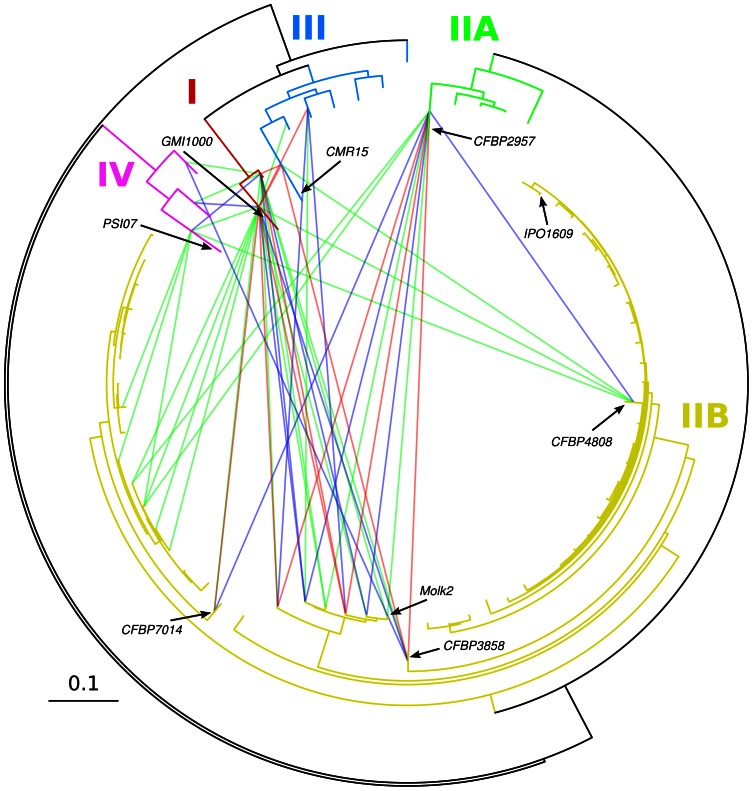
Putative highways of HGT. Circular representation of the phylogenetic tree based on the hybridization of 7,055 probes from the six fully sequenced strains. Putative horizontal gene transfer are represented using lines between tips of the tree. The lines are colored according to the significance level of having more gene sharing than expected by chance with green, blue and red for *p*-values superior to 0.99, 0.999 and 0.9999 respectively. Branches are colored according to the phylotype classification.

As the high mobility of genes tends to support high HGT frequency between strains, we devised a simple procedure to detect putative HGT. Using the gain data, we flagged pairs of strains displaying more genes in common but absent in their last common ancestor than expected by chance (Figure 5). Most of the putative HGT events were detected between strains grouping with the Molk2 strain (sequevar 3 strains [47] from the phylotype IIB) and both phylotype I, and phylotype IIA. A restricted number of HGT events were apparent between phylotypes I, III and IV. In previous work, recombination between strains was inferred from MLSA data, and different patterns of exchanged were obtained particularly with the IIB strains appearing isolated [48]. While homologous recombination and HGT are two distinct processes, one can expect the same limitations and patterns of exchange to have come out of these two analyses. While in the former study, only nine genes were compared, it is important to notice here that due to the data we analysed (gene presence/absence), only the most obvious fraction of the HGTs were probably detected in this current study. Also, the network of gene exchange clearly bore marks of the CGH microarray design since the sequenced strains (exception of IPO1609) were involved in most of the detected HGTs.

Some of the strains, namely CFBP4808 and CFBP3858, displayed a high number of HGT events and a large number of genes present in only one of the six fully sequenced strains (Figure S4). These features explain their positions on the phylogenetic tree as outliers (CFBP3858) or with a long terminal branch (CFBP4808). As recombination is known to confound phylogeny (HGT will impact the binary matrix of presence/absence exactly as recombination would do on a DNA sequence alignment), their positioning highlights the difficulty to reconstruct the phylogenies of strains with reticulate evolutionary past. Interestingly, strain-specific genes from all the six sequenced strains were present in high proportions in their genomes (see Figure S4). Although consistent with the propensity of *R. solanacearum* to take up and recombine exogenous DNA (up to 30 Kb of contiguous DNA *in planta* [60]–[62]), whether these gene transfers occurred *in planta* or not and directly between *R. solanacearum* strains or from free DNA fragments remains difficult to identify.

Interestingly, these two strains belong to the phylotype IIB, a phylotype that probably emerged in South America [48], [63] but was isolated in Israel (CFBP4808) and the Netherlands (CFBP3858). They may have had the opportunity to acquire genes from other distinct groups in those locations. In fact the region of origin and the region of diversification may not overlap. Identifying key reservoir species or geographic areas from which gene transfers originated would likely help to predict the impact of human activity such as agriculture and trade, on the emergence of new pathogens.

### Concluding remarks

The pan-genomic microarray approach, with its high throughput capability, provided us with the opportunity to assess the gene content of a large number of strains and to reconstruct the history of gene loss and acquisitions. The pan-genome of *R. solanacearum* is extremely large with almost 70% of genes considered as accessory, and it is interesting to note that it is also highly variable between strains. While gene presence/absence represents only a subset of the sequence variation between strains, we believe it is informative to analyze the dynamics of gene presence/absence, loss and gain in order to understand one of the major layers of bacterial genome evolution. It was clearly apparent that factors such as gene function and gene localization are important in determining gene transferability. While it has been hypothesized that the distinct phylotypes may have evolved in different species [31], gene flow between phylotypes may indicate that speciation is not achieved [64]. Further studies, intending to examine the genome fluidity and precise conditions that favor possible gene transfer would provide invaluable insights into species delineation and the emergence of successful pathogens.

## Supporting Information

Figure S1 **CGH microarrays validation on R229 and UW551.** Plot of the false-positive (blue), and false-negative (blue) calls from the CGH-microarrays on the UW551 (solid lines) and R229 (dashed lines) genomes depending on the cutoff used to define homology. (B) Pairwise homology of the CDS from the six sequenced genomes targeted by the same probe.
(TIFF)

Figure S2 **Phylogenetic tree based on the 2,992 blocks of probes.** Phylogenetic tree of the *R. solanacearum* species complex inferred using MrBayes and based on the hybridization results of 2,992 blocks of contiguous probes in the genomes and display the same evolutionary patterns. Strains used for the construction of the microarrays are in bold. Phylotype classification is indicated using colored rectangles. Black circles on nodes indicate posterior probability branching support superior to 95%.
(TIFF)

Figure S3 **Cold-spots and hot-spots of gene movement.** Schematic representation of the cold-spots and hot-spots of gene movement along the genomes of the GMI1000, CFBP2957, CMR15 and PSI07 strains. Cold-spots are indicated in blue while hot-spots are indicated in red. Putative IS elements are represented with purple lines. On every genome, two tests (*p*-value <10^−4^) are represented. For each test, gene movement patterns were compared to those obtained after 10,000 permutations of gene order using a sliding window of 100 (A), 200 (B), 400 (C) and 1,000 (D) genes. In the first “global” test, permutations were performed over the concatenated chromosome and megaplasmid. This test was designed to detect differential patterns between both components. In the second “local” test, permutations were performed on each genomic component separately. This second test was designed to detect intra-component patterns of differential gene movement.
(TIFF)

Figure S4 **Distribution of the genes specific to the six sequences strains.** Phylogenetic tree of the *R. solanacearum* species complex along with the per-strain proportion of genes targeted by probes designed as specific to the GMI1000, CFBP2957, Molk2, IPO1609, CMR15 and PSI07 strains.
(TIFF)
