# Supplementary figures and images for: Constraints on Genome Dynamics Revealed from Gene Distribution among the Ralstonia solanacearum Species
Source: PLoS One. 2013 May 28;8(5):e63155. doi: 10.1371/journal.pone.0063155 (PMC3665557; doi:10.1371/journal.pone.0063155)

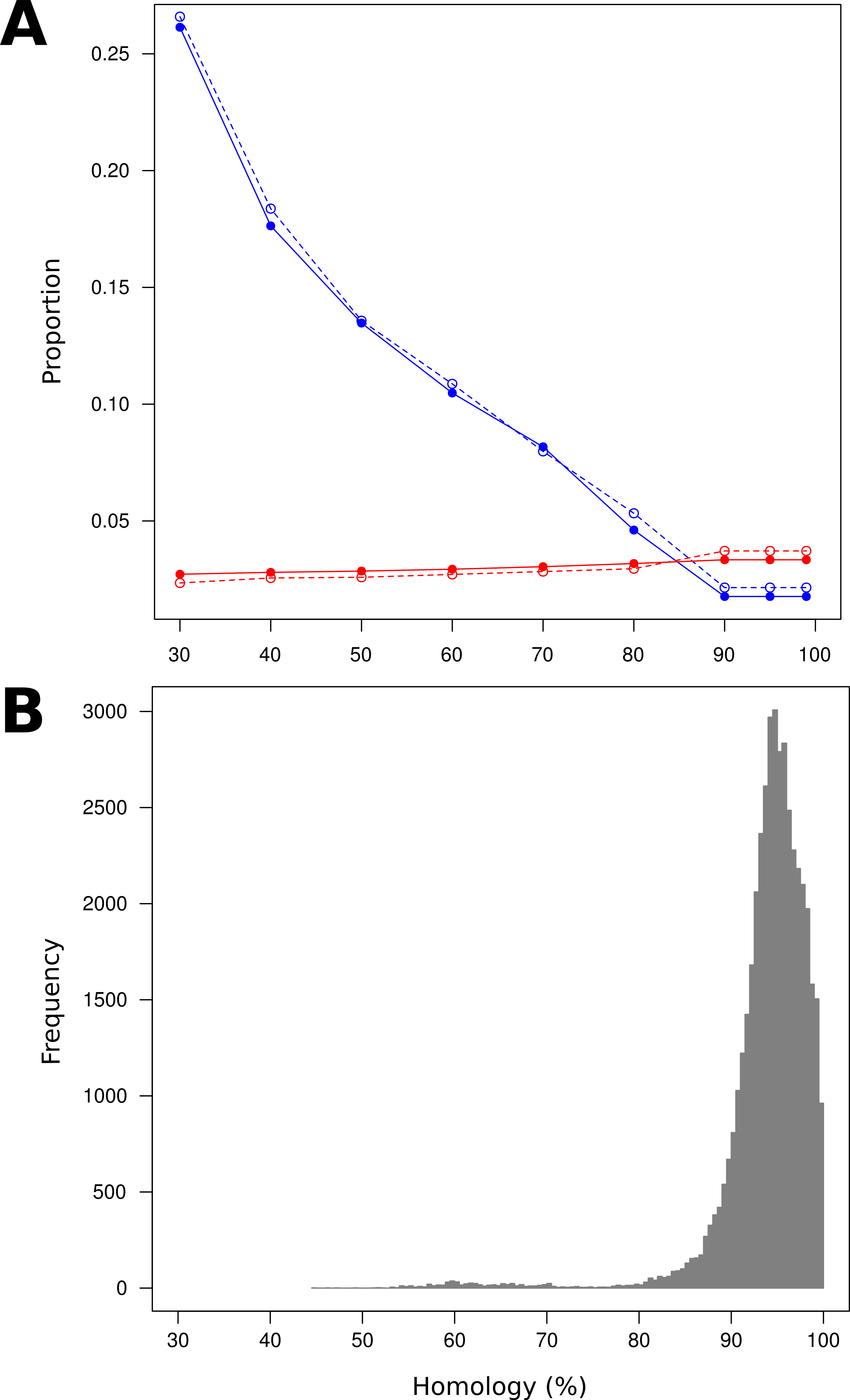

Supplement: Figure S1 — CGH microarrays validation on R229 and UW551. Plot of the false-positive (blue), and false-negative (blue) calls from the CGH-microarrays on the UW551 (solid lines) and R229 (dashed lines) genomes depending on the cutoff used to define homology. (B) Pairwise homology of the CDS from the six sequenced genomes targeted by the same probe. (TIFF) [file pone.0063155.s001.tiff]

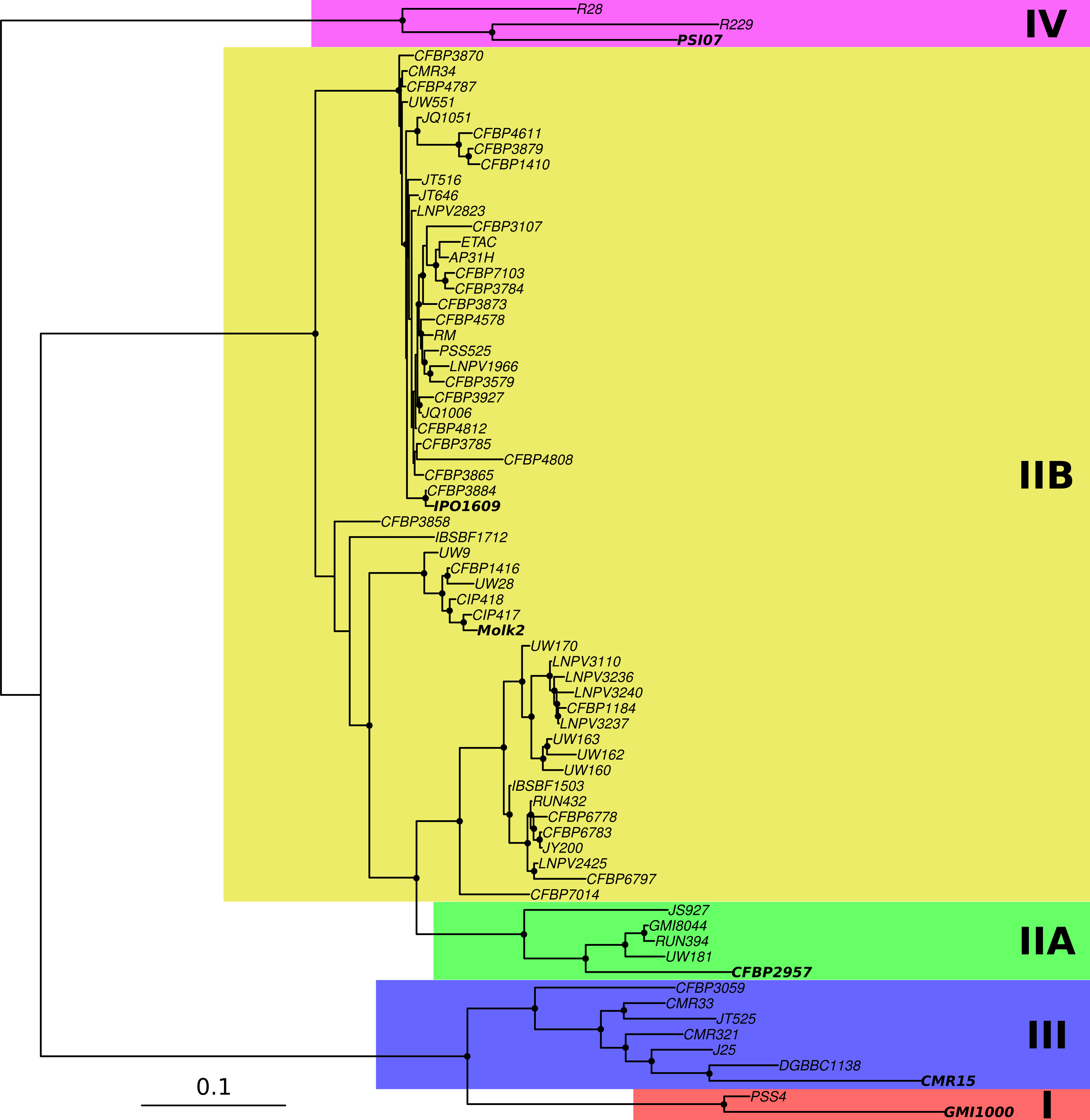

Supplement: Figure S2 — Phylogenetic tree based on the 2,992 blocks of probes. Phylogenetic tree of the R. solanacearum species complex inferred using MrBayes and based on the hybridization results of 2,992 blocks of contiguous probes in the genomes and display the same evolutionary patterns. Strains used for the construction of the microarrays are in bold. Phylotype classification is indicated using colored rectangles. Black circles on nodes indicate posterior probability branching support superior to 95%. (TIFF) [file pone.0063155.s002.tiff]

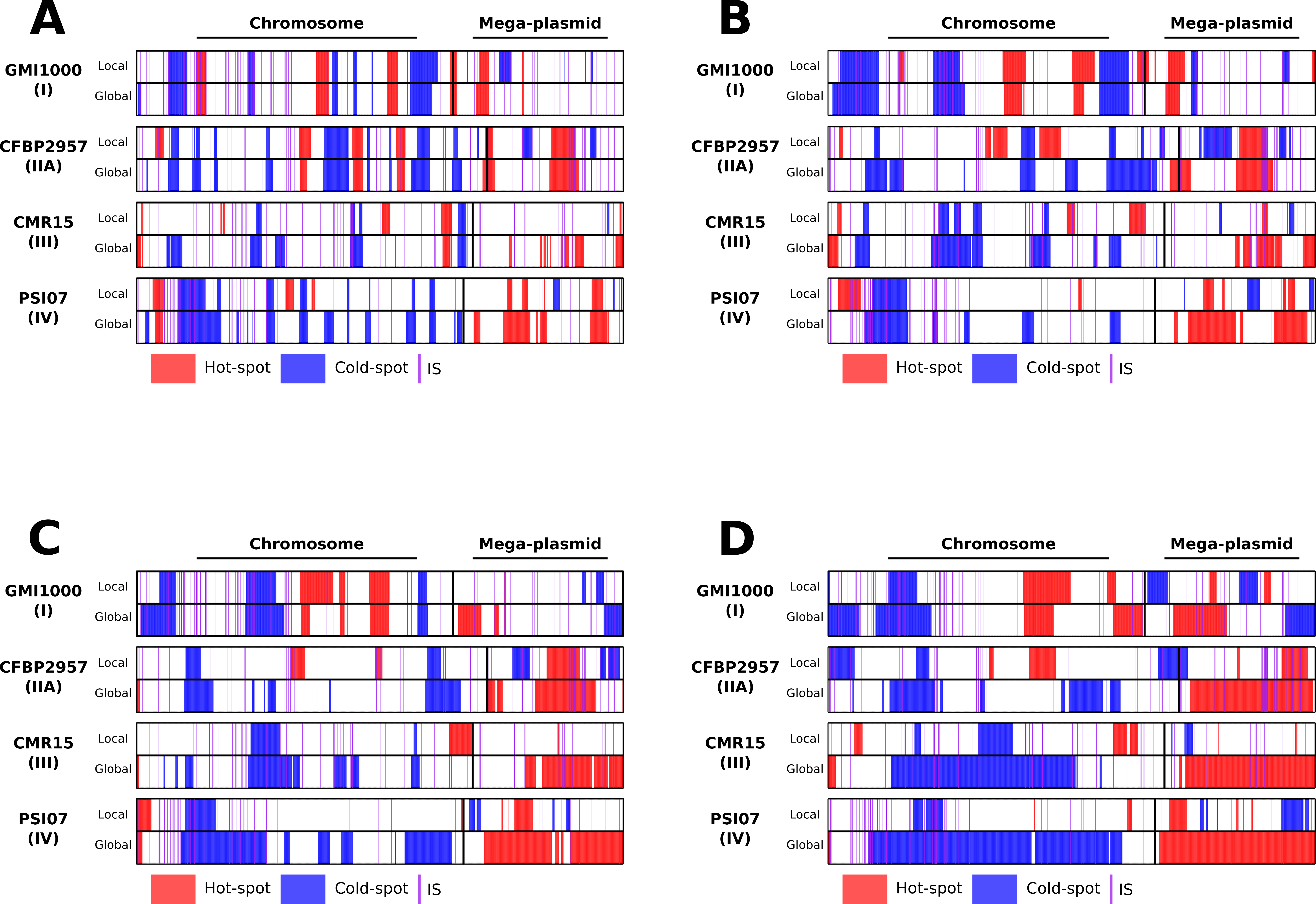

Supplement: Figure S3 — Cold-spots and hot-spots of gene movement. Schematic representation of the cold-spots and hot-spots of gene movement along the genomes of the GMI1000, CFBP2957, CMR15 and PSI07 strains. Cold-spots are indicated in blue while hot-spots are indicated in red. Putative IS elements are represented with purple lines. On every genome, two tests (p-value <10−4) are represented. For each test, gene movement patterns were compared to those obtained after 10,000 permutations of gene order using a sliding window of 100 (A), 200 (B), 400 (C) and 1,000 (D) genes. In the first “global” test, permutations were performed over the concatenated chromosome and megaplasmid. This test was designed to detect differential patterns between both components. In the second “local” test, permutations were performed on each genomic component separately. This second test was designed to detect intra-component patterns of differential gene movement. (TIFF) [file pone.0063155.s003.tiff]

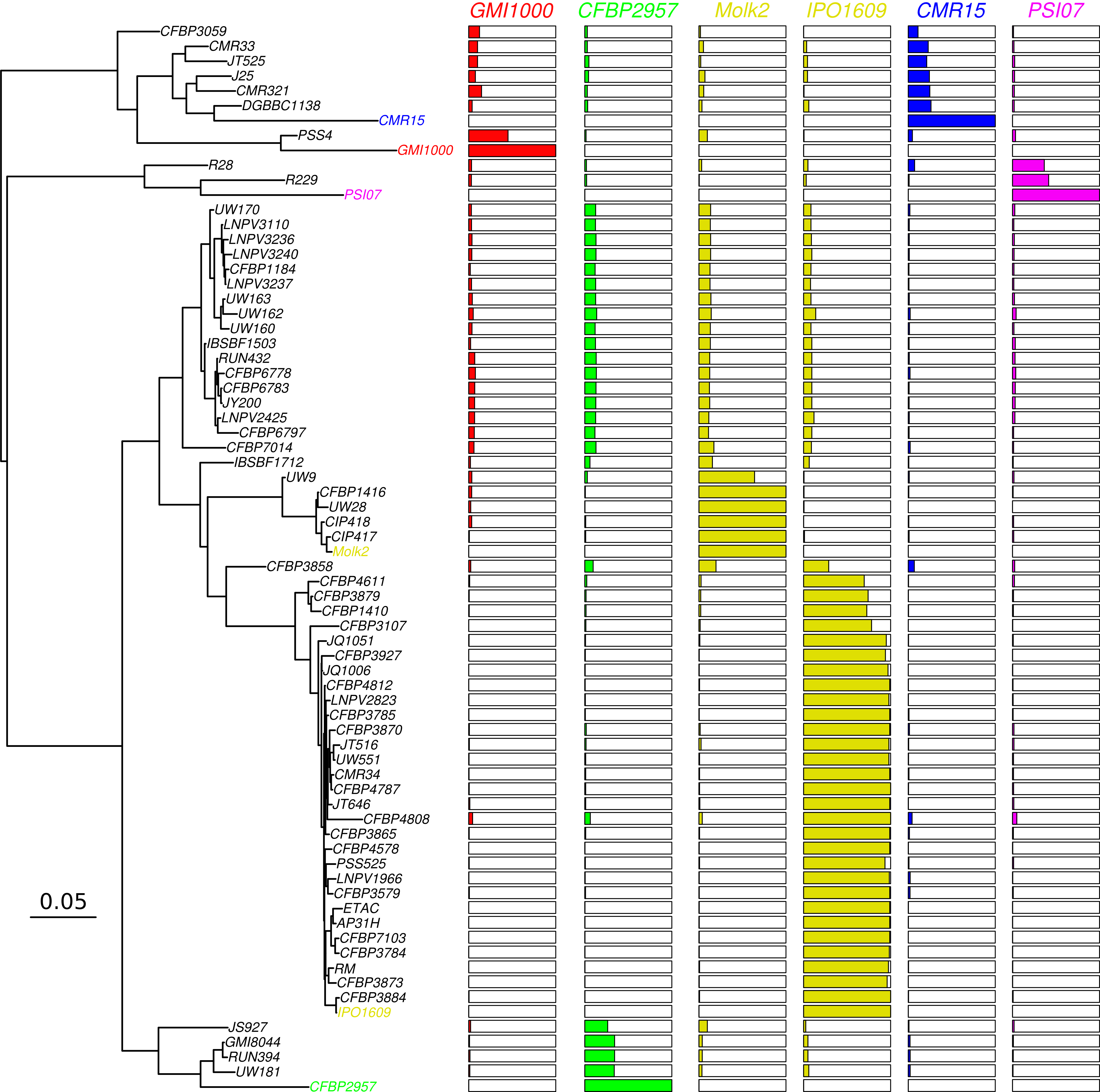

Supplement: Figure S4 — Distribution of the genes specific to the six sequences strains. Phylogenetic tree of the R. solanacearum species complex along with the per-strain proportion of genes targeted by probes designed as specific to the GMI1000, CFBP2957, Molk2, IPO1609, CMR15 and PSI07 strains. (TIFF) [file pone.0063155.s004.tiff]
